# Supplementary material for: Large evanescently-induced Brillouin scattering at the surrounding of a nanofibre
Source: Nat Commun. 2022 Mar 17;13:1432. doi: 10.1038/s41467-022-29051-8 (PMC8931049; doi:10.1038/s41467-022-29051-8)
Supplement: Supplementary file 1 — Supplementary Information [file 41467_2022_29051_MOESM1_ESM.pdf]

Supplementary Information for: Large evanescently-induced Brillouin scattering at the surrounding of a nanofibre

Fan Yang[1,4,*], Flavien Gyger[1,5], Adrien Godet[2], Jacques Chrétien[2], Li Zhang[1], Meng Pang[3], Jean-Charles Beugnot[2,*], and Luc Thévenaz[1]

[1]Ecole Polytechnique Fédérale de Lausanne (EPFL), Group for Fibre Optics, CH-1015 Lausanne, Switzerland

[2]Institut FEMTO-ST, UMR 6174, Université Bourgogne Franche-Comté, 25030 Besançon, France

[3]State Key Laboratory of High Field Laser Physics, Shanghai Institute of Optics and Fine Mechanics, CAS, Shanghai 201800, China

[4]Present address: European Molecular Biology Laboratory, Heidelberg, Germany

[5]Present address: Max Planck Institute of Quantum Optics, Garching, Germany

* Corresponding author: *fanyang808@gmail.com*,

jean-charles.beugnot@femto-st.fr

Supplementary Note 1 Numerical simulation of Brillouin scattering in nanofibre surrounding by 40 bar CO_2

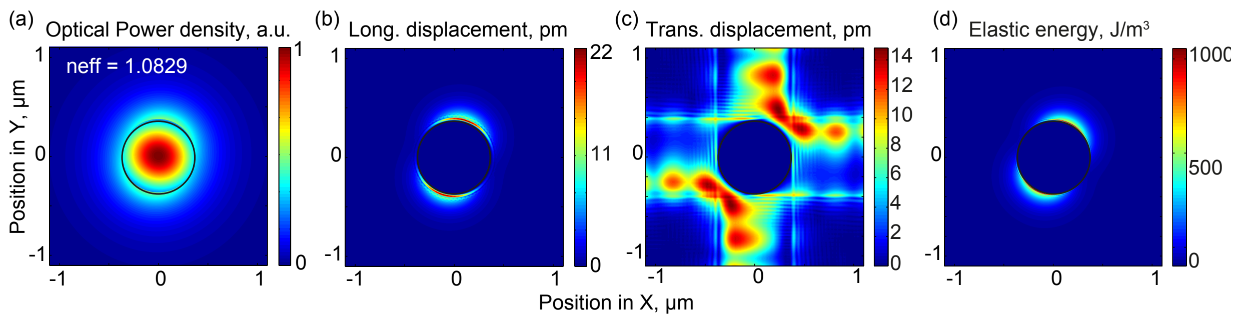

Supplementary Figure 1: **Numerical simulation of Brillouin scattering in nanofibre surrounding by 40 bar CO_2.** (a) Optical power density of the fundamental TE-like mode for pump wavelength of 1550 nm. Spatial distribution of longitudinal (b) and transverse (c) displacement, respectively. (d) Spatial distribution of elastic energy density of resonant phonon in CO_2 at 350 MHz.

Supplementary Figure 1 shows the numerical simulation results of Brillouin scattering in a 740 nm diameter nanofibre surrounding by 40 bar CO_2. The optical power density is plotted in Supplementary Figure 1(a). The longitudinal and transverse displacement are shown in Supplementary Figure 1(b) and Supplementary Figure 1(c) respectively. The spatial distribution of the elastic energy density is shown in Supplementary Figure 1(d). The elastic wave generated by the optical evanescent field in the CO_2 around the nanofibre is confined to the boundary of the nanofibre. The most important contribution of the elastic wave comes from longitudinal displacement and the transverse displacement has negligible contribution to the elastic wave.

Supplementary Note 2 Calculation of the Brillouin gain coefficient

The Brillouin gain coefficient and spectrum can be obtained by the spontaneous Brillouin scattering measurement [1]. It has also been used for forward Brillouin scattering characterisation [2, 3]. The Brillouin gain can be represented by $G = g_B P_{pump} L$, where g_B is the Brillouin gain coefficient in $m^{-1}W^{-1}$, P_{pump} is the pump optical power input to the fibre, and L is the fibre length. When $G \ll 1$, which is the case when the reflected light originates entirely from the scattering of the laser field by spontaneously (i.e., thermally) generated phonons, the total generated Stokes and anti-Stokes optical power is given by [1]:

$$P_{\mathrm{Stokes}} \cong P_{\mathrm{anti-Stokes}} \equiv \frac{\pi}{2} g_{\mathrm{B}} P_{\mathrm{pump}} L(\overline{n}+1) h\nu_{\mathrm{pump}}\delta_{\nu} \tag{1}$$

where P_{Stokes} and $P_{\mathrm{anti-Stokes}}$ are the total Stokes and anti-Stokes optical power, $\overline{n} = (e^{\frac{h\nu_B}{KT}} - 1)^{-1}$ is the mean number of phonons per mode of the acoustic field, h is the Planck constant, ν_B is the Brillouin frequency shift (i.e. resonant acoustic frequency), K is the Boltzmann constant, T is the temperature in K, ν_{pump} is the pump frequency, δ_ν is the full-width half-maximum Brillouin linewidth.

The reflected spontaneous Stokes and anti-Stokes spectrum are measured by the set-up shown in Fig. 4(a) in the main manuscript. The Stokes and anti-Stokes power are calibrated by beating a known power distributed feedback (DFB) laser with an external cavity diode laser (ECDL) and the calibration set-up is shown in Supplementary Figure 2(a). In Fig. 4(a) in the main manuscript, we measure not only the evanescent Brillouin scattering in the nanofibre gas cell (beating frequency of the Stokes signal at $\sim$ 500 MHz) but also the Brillouin scattering in standard single-mode fibre (beating frequency of the Stokes signal at $\sim$ 10.9 GHz). In order to measure the different response of the photo-detector (Newport model 1544) at 500 MHz and 10.9 GHz, we tune the frequency difference between the DFB laser and the ECDL at these two frequency regions. The beating spectra at these two frequency regions are shown in Supplementary

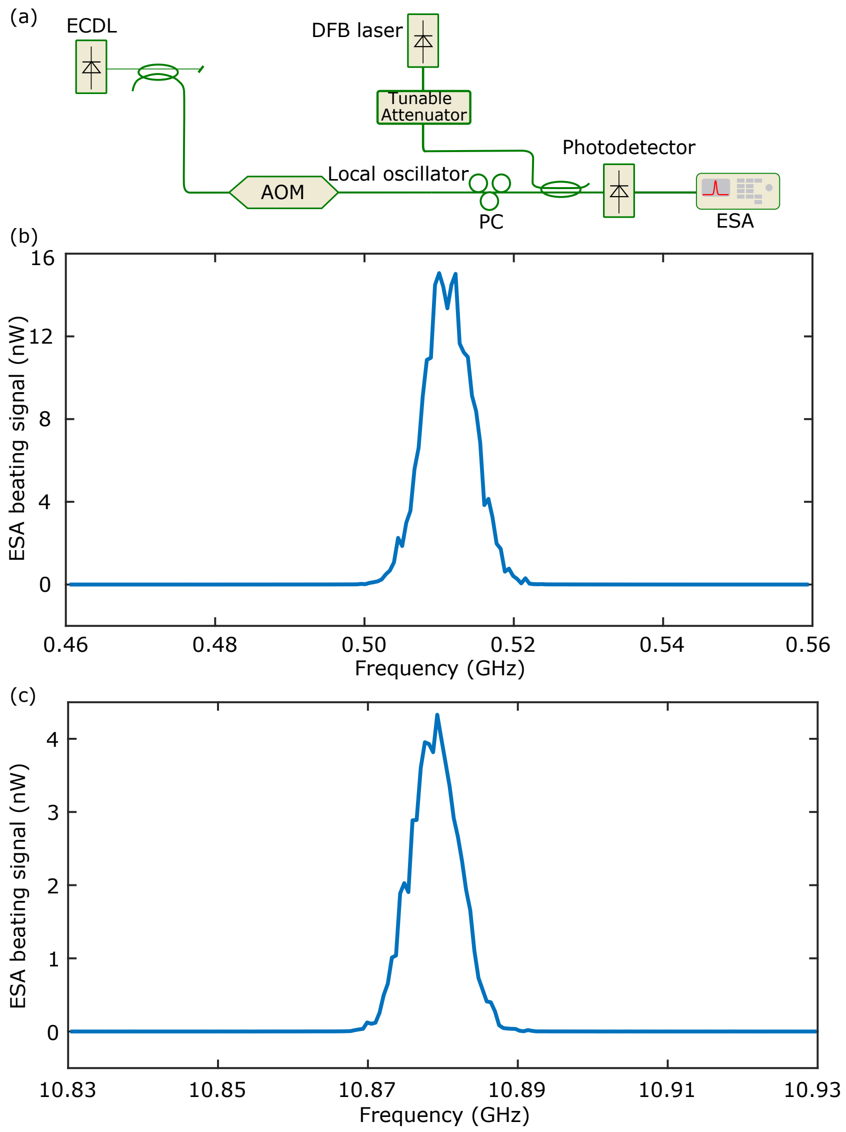

Supplementary Figure 2: **Experimental beating signal between a distributed-feedback (DFB) laser and an external-cavity diode laser (ECDL).** (a) The beating experimental set-up. A DFB laser with power of -40 dBm (tuned by a tunable attenuator) beats with the ECDL which is used in the main manuscript. The beating signal is measured by a photodetector and an electrical spectrum analyser (ESA). The polarisation controller (PC) is used to maximise the beating signal. The resolution bandwidth and video bandwidth of the ESA are both 1 MHz. (b) Beating electrical power signal at low frequency range ($\sim$ 500 MHz) and (c) high frequency range ($\sim$ 10.9 GHz).

Figs. 2(b) and (c) respectively with the attenuator tuned such that the DFB laser light reaches the coupler with a power of -40 dBm. The reflected total Stokes and anti-Stokes power due to gas evanescent Brillouin scattering in the 10 cm nanofibre gas cell are 4.5 nW with a pump power of 90 mW. The reflected total Stokes and anti-Stokes power due to Brillouin scattering in the 53.5 m standard single-mode fibre (SMF) are 2.9 nW with a pump power of 80 mW. Note the pump power difference is due to the insertion loss of the nanofibre. The measured linewidth for the gas evanescent Brillouin scattering and SMF Brillouin scattering are 17.1 MHz and 25.3 MHz respectively. With all these parameters, the peak Brillouin gain coefficient for the nanofibre gas cell filled with 40 bar CO_2 and for the SMF are calculated to be 8.2 m $^{-1}$W^{-1} and 0.24 m^{-1}W^{-1} respectively. The estimated SMF Brillouin gain coefficient 0.24 m^{-1}W^{-1} is in excellent agreement

with the value known for the SMF [4].

Supplementary Note 3 Analysis of the Brillouin signal from the nanofibre and tapered regions

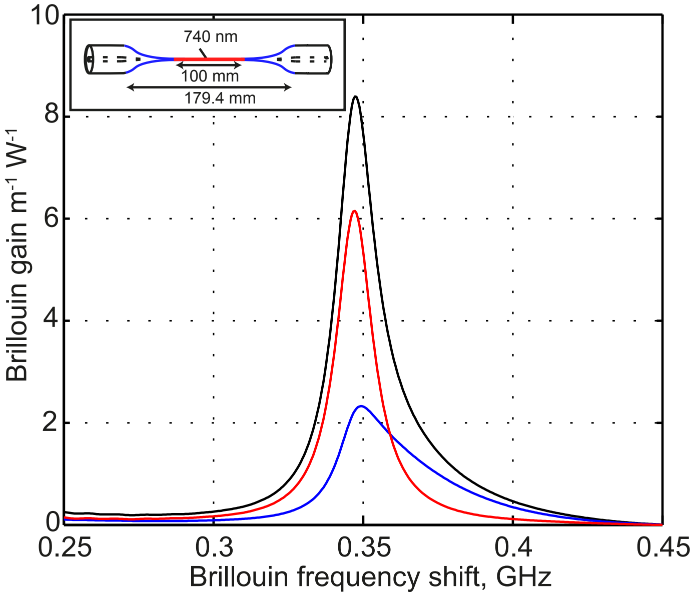

Supplementary Figure 3: **Theoretical Brillouin spectra in 40 bar CO_2 obtained in a nanofibre with a waist of 740 nm.** The red line shows the theoretical Brillouin spectrum at the uniform nanofibre waiste region; the blue line shows the tapered regions; the black line shows the total Brillouin spectrum.

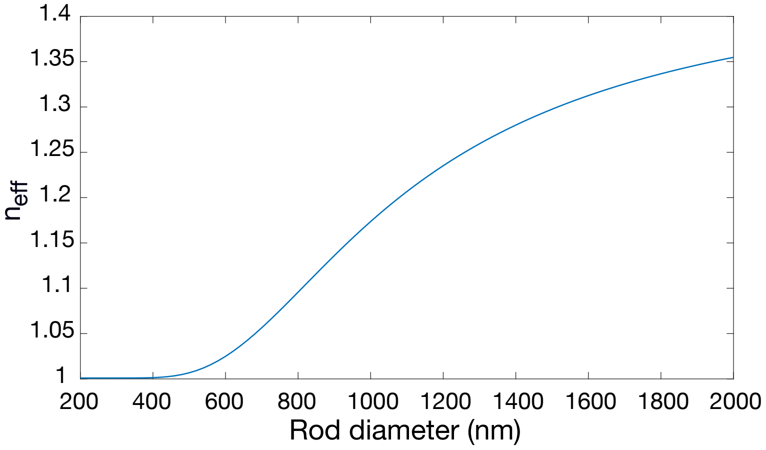

Supplementary Figure 4: **Effective refractive index as a function of rod diameter.**

Supplementary Figure 3 shows the different contributions of a nanofibre on the Brillouin spectrum. The red and blue lines are the theoretical Brillouin spectrum at the uniform nanofibre waist region and tapered region, respectively. The black line is the total Brillouin spectrum. Supplementary Figure 4 illustrates the effective refractive index as a function of the rod diameter. The resonant Brillouin frequency shift

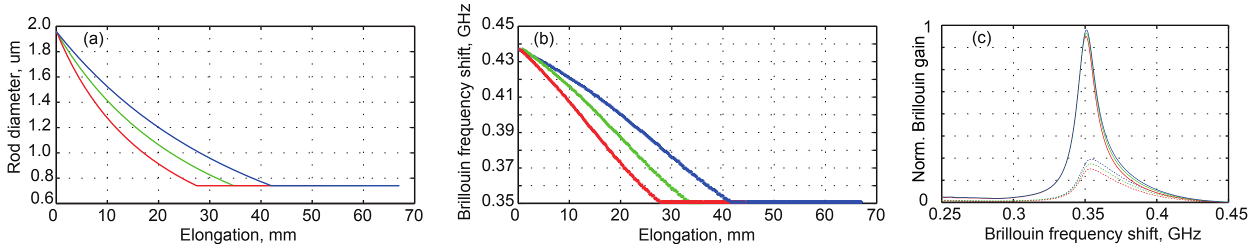

Supplementary Figure 5: **Simulation results with different tapered profile.** The rod diameter (a) and the Brillouin frequency shift (b) as a function of the elongation for three tapered profiles. (c) The normalised Brillouin spectra for three tapered profiles shown in (a). Solid lines show the total Brillouin spectra and dotted lines show the contribution from the tapered regions for three tapered profiles.

$\nu_B = 2n_{eff}V_a/\lambda$, where n_{eff} is the effective refractive index of the optical mode, V_a is the acoustic velocity of the surrounding gas and λ is the pump wavelength in vacuum. Since the tapered regions have larger diameter than the waist region, the effective refractive index in the tapered regions is larger than in the waist region. Therefore, the Brillouin frequency shift in the tapered regions is larger than in the waist which is shown in Supplementary Figure 3.

Our taper fabrication process is based on 3 steps to obtain an adiabatic transmission and a very low loss [5]. The length of the adiabatic transition region is estimated by subtracting the total length of tapered fibre and the length of uniform section. In our case, the length of homogeneous section is 100 mm and the total length of tapered optical fibre is 179.4 mm. This value is precise because it is given by the translation stage. The length of both adiabatic transition regions is consequently 79.4 mm $\pm$ 5 mm. The very good agreement between integrated measurement and calculation of Brillouin scattering all along the length of tapered optical fibre represented in Fig. 5(b) in the main manuscript reinforces the estimation of nanofibre length. The distributed Brillouin measurement in silica tapered optical fibre demonstrates the good agreement between estimation and measurements of the different lengths of tapered optical fibre [6].

As shown in Supplementary Figure 5, we simulate the Brillouin spectra with different tapered profile. As we can see in Supplementary Figure 5(c), the tapered profile has little impact on the left side of the Brillouin spectrum.

Supplementary Note 4 Brillouin frequency shift as a function of CO_2 pressure in the nanofibre gas cell

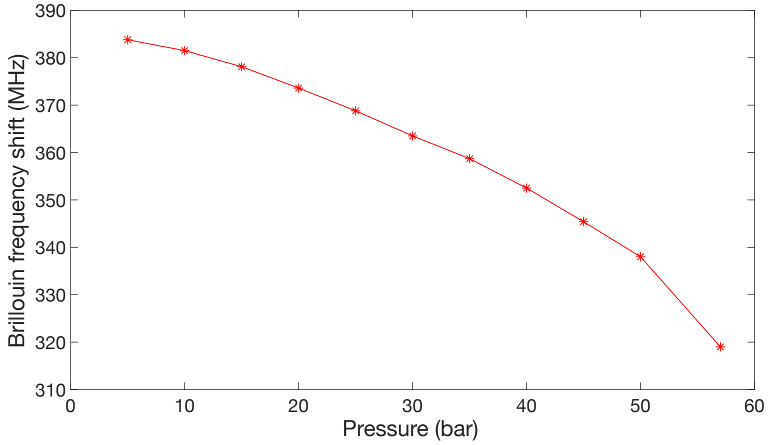

Supplementary Figure 6: **Experimental Brillouin frequency shift for the nanofibre gas cell filled with different pressure of CO_2 gas.**

Supplementary Figure 6 shows the experimental Brillouin frequency shift for the nanofibre gas cell filled with different pressures of CO_2 gas. It demonstrates that our platform can be used for CO_2 gas pressure measurement by measuring the Brillouin frequency shift. Note that it can also be used for temperature measurement because for a fixed pressure, the acoustic velocity in the gas and hence the Brillouin frequency shift is related with the gas temperature with a coefficient of about 1.2 MHz/K.

Supplementary Note 5 Theoretical analysis of the Brillouin scattering in a nanofibre liquid cell

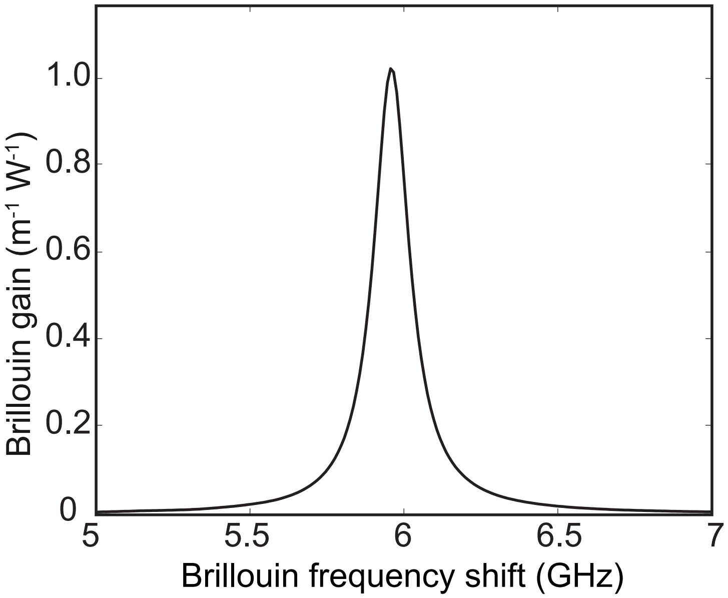

Supplementary Figure 7: **Numerical calculation of evanescent Brillouin scattering in silica nanofiber surrounded by water.**

The Brillouin scattering generated by the evanescent field of a nanofibre in water is calculated and shown in Supplementary Figure 7. In the simulation, we use the parameters from [7]. The optimal diameter of the nanofibre maximising the Brillouin scattering by the evanescent field in water is calculated to be 450 nm. The pump wavelength is 694 nm. The Brillouin gain coefficient of double-distilled water in units of m/W is 7.53×10^{-11} m/W at this wavelength [8]. The Brillouin frequency shift and Brillouin linewidth in water are 5.91 GHz and 371 MHz, respectively. In the calculation, the contribution from tapered regions is not included.

The peak Brillouin gain in Supplementary Figure 7 is calculated to be 1 $m^{-1}W^{-1}$ which is equal to the peak Brillouin gain of a 740 nm nanofibre surrounded with 10 bar CO_2 and is 4 times higher than that of the standard single-mode fibre. This result shows good capability of our nanofibre waveguide for Brillouin spectroscopy and microscopy.

In order to make our calculation more convincing, here we do an intuitive estimation using the measured results by previous works. The measured Brillouin coefficient of 40 bar CO_2 at 1550 nm is 1.34×10^{-10} m/W [9]. In our current work, the measured Brillouin gain coefficient of a nanofibre filled with 40 bar CO_2 (in units of $m^{-1}W^{-1}$) at 1550 nm is 8.2 $m^{-1}W^{-1}$. So we estimated the Brillouin gain coefficient of water (in units of $m^{-1}W^{-1}$) to be 3.6 $m^{-1}W^{-1}$. This estimated value is 3.6 times larger than our simulated value which may result from the larger mode area in a nanofibre surrounded with water compared to a nanofibre surrounded with 40 bar CO_2 gas.

Supplementary References

[1] Boyd, R. W., Rzazewski, K. & Narum, P. Noise initiation of stimulated Brillouin scattering. *Physical Review A* **42**, 5514 (1990). URL https://journals.aps.org/pra/abstract/10.1103/PhysRevA.42.5514.

[2] Renninger, W. H. *et al.* Forward Brillouin scattering in hollow-core photonic bandgap fibers. *New Journal of Physics* **18**, 025008 (2016). URL https://doi.org/10.1088/1367-2630/18/2/025008.

[3] Renninger, W. H., Behunin, R. O. & Rakich, P. T. Guided-wave Brillouin scattering in air. *Optica* **3**, 1316–1319 (2016). URL https://www.osapublishing.org/optica/abstract.cfm?uri=optica-3-12-1316.

[4] Motil, A., Bergman, A. & Tur, M. [INVITED] State of the art of Brillouin fiber-optic distributed sensing. *Optics & Laser Technology* **78**, 81–103 (2016). URL https://www.sciencedirect.com/science/article/pii/S0030399215002571.

[5] Hoffman, J. E. *et al.* Ultrahigh transmission optical nanofibers. *AIP Advances* **4**, 067124 (2014). URL https://doi.org/10.1063/1.4879799.

[6] Chow, D. M. *et al.* Local activation of surface and hybrid acoustic waves in optical microwires. *Opt. Lett.* **43**, 1487–1490 (2018). URL http://ol.osa.org/abstract.cfm?URI=ol-43-7-1487.

[7] Brignon, A. & Huignard, J. P. *Phase Conjugate Laser Optics* (Wiley, 2003).

[8] Remer, I., Shaashoua, R., Shemesh, N., Ben-Zvi, A. & Bilenca, A. High-sensitivity and high-specificity biomechanical imaging by stimulated Brillouin scattering microscopy. *Nature Methods* **17**, 913–916 (2020). URL https://www.nature.com/articles/s41592-020-0882-0.

[9] Yang, F., Gyger, F. & Thévenaz, L. Intense Brillouin amplification in gas using hollow-core waveguides. *Nature Photonics* **14**, 700–708 (2020). URL https://www.nature.com/articles/s41566-020-0676-z.
